# Using record linkage to validate notification and laboratory data for a more accurate assessment of notifiable infectious diseases

**DOI:** 10.1186/s12911-017-0484-7

**Published:** 2017-06-17

**Authors:** Faye J. Lim, Christopher C. Blyth, Avram Levy, Parveen Fathima, Nicholas de Klerk, Carolien Giele, Hannah C. Moore

**Affiliations:** 1 Wesfarmers Centre of Vaccines and Infectious Diseases, Telethon Kids Institute, The University of Western Australia, PO Box 855, West Perth, WA 6872 Australia; 2 School of Paediatrics and Child Health, The University of Western Australia, GPO Box D184, Perth, WA 6840 Australia; 3 Department of Infectious Diseases, Princess Margaret Hospital for Children, GPO Box D184, Perth, WA 6840 Australia; 4 PathWest Laboratory Medicine WA, QE2 Medical Centre, Locked Bag 2009, Nedlands, WA 6906 Australia; 5 School of Pathology and Laboratory Medicine, M504, The University of Western Australia, 35 Stirling Highway, Crawley, WA 6009 Australia; 6 Communicable Disease Control Directorate, Western Australian Department of Health, PO Box 8172, Perth Business Centre, Perth, 6879 Australia

**Keywords:** Disease notification, Record linkage, Validation studies

## Abstract

**Background:**

Infectious disease burden is commonly assessed using notification data. Using retrospective record linkage in Western Australia, we described how well notification data captures laboratory detections of influenza, pertussis and invasive pneumococcal disease (IPD).

**Methods:**

We linked data from the Western Australian Notifiable Infectious Diseases Database (WANIDD) and the PathWest Laboratory Database (PathWest) pertaining to the Triple I birth cohort, born in Western Australia in 1996–2012. These were combined to calculate the number of unique cases captured in each dataset alone or in both datasets. To assess the impact of under-ascertainment, we compared incidence rates calculated using WANIDD data alone and using combined data.

**Results:**

Overall, there were 5550 influenza, 513 IPD (2001–2012) and 4434 pertussis cases (2000–2012). Approximately 2% of pertussis and IPD cases and 7% of influenza cases were solely recorded in PathWest. Notification of influenza and pertussis cases to WANIDD improved over time. Overall incidence rates of influenza in children aged <5 years using both datasets was 10% higher than using WANIDD data alone (IRR = 1.1, 95% CI = 1.1–1.2).

**Conclusions:**

This is the first time WANIDD data have been validated against routinely collected laboratory data. We anticipated all cases would be captured in WANIDD but found additional laboratory-confirmed cases that were not notified. Studies investigating pathogen-specific infectious disease would benefit from using multiple data sources.

**Electronic supplementary material:**

The online version of this article (doi:10.1186/s12911-017-0484-7) contains supplementary material, which is available to authorized users.

## Background

Population estimates of the burden of some infectious diseases may be calculated using surveillance data based on statutory notifications. In Australia, a disease is listed on the National Notifiable Diseases list if it is a public health priority and data collection is feasible [1]. Data on these diseases are collected by surveillance or other methods at individual state and territory Departments of Health and subsequently sent to the national Department of Health for national surveillance [2].

While automated notification-based surveillance systems are cost-effective, timely and ease the reporting burden on healthcare providers, if these systems are not audited and updated regularly, incomplete data capture may occur with changes over times (e.g. changes in case definitions or testing methods). These may lead to inaccurate estimates of disease burden. In Western Australia (WA), we have the opportunity to use record linkage (also known as data linkage) to assess the completeness of data capture using these systems. Record linkage is the process of combining multiple, usually administrative, datasets that relate to the same person, and has been used successfully in epidemiological studies of infectious diseases in Australia and the United Kingdom [3–5].

Infectious diseases, particularly acute respiratory infections, are the leading cause of hospitalisation in young children in WA [6]. Influenza, pertussis (whooping cough) and invasive pneumococcal disease (IPD) are notifiable infectious diseases in WA, which are usually associated with pathogens causing respiratory infections. In Australia, pertussis became notifiable in 1991 while influenza and IPD became notifiable in 2001 [7, 8].

In WA, the responsibility for notification lies with the attending healthcare provider [9]. In addition, diagnostic laboratories responsible for notifiable disease testing are also required to report detections of such pathogens [9]. Both groups are encouraged to complete a disease notification regardless of whether the case has been reported previously by another party, with duplicates managed by the WA Department of Health [9]. These data are stored on the Western Australian Notifiable Infectious Diseases Database (WANIDD) [10].

As part of a whole population-based birth cohort study investigating the pathogen-specific burden of respiratory infections in children (referred to as the Triple I cohort), we assembled a linked dataset of WANIDD detections and routine laboratory data from the PathWest Laboratory Database using the WA Data Linkage System. PathWest Laboratory Medicine is a state-wide public laboratory that provides pathology testing services to all public hospitals in WA, some general practitioners from private clinics, and referred samples from private pathology providers [4, 11]. Approximately half of the specimens tested at PathWest Laboratory Medicine are from children presenting to general practices or outpatient clinics, with the remainder from children admitted to hospital (personal communication, A Levy). The PathWest Laboratory Database stores all data relating to pathology testing conducted at PathWest Laboratory Medicine. When pathogens associated with notifiable diseases are reported in the PathWest Laboratory Database (PathWest), a report of these cases is sent automatically to WANIDD.

We assess the completeness of data capture in WANIDD by comparing recorded detections in PathWest. We wanted to describe the differences, if any, in the number of cases with notifiable diseases recorded in WANIDD compared with PathWest, as well as to identify the effects of these differences on estimates of laboratory-confirmed infections. To do this, we focused on three key infections: laboratory-proven influenza, *Bordetella pertussis*, and invasive *Streptococcus pneumoniae* infection (associated with IPD). Given the statutory requirements for notification of these infections and the automated reporting system in PathWest, we anticipated that all PathWest detections of these pathogens would also be captured in WANIDD. As a proportion of diagnostic samples are tested only by private laboratories, we expected detections in these laboratories to be captured solely in WANIDD.

## Methods

The Triple I cohort included all children born in WA between 1996 and 2012. The Triple I cohort was identified using data extracted from the Midwives Notification System and the Birth and Death Registry via the WA Data Linkage System. An estimated 95% of children in this study were successfully linked across datasets through this system (personal communication, WA Data Linkage Branch). The Triple I cohort comprised of 469,589 children, of whom 51.2% were male and 6.7% were of Aboriginal or Torres Strait Islander descent (hereafter referred to as Aboriginal). The majority of these children (74.7%) were born in metropolitan Perth.

PathWest and WANIDD records of children in the Triple I cohort that reported laboratory-proven influenza virus infection, *B. pertussis* or invasive *S. pneumoniae* infections were extracted for this study. Comparisons were restricted to data with a date of specimen collection between January 2000 and December 2012. For WANIDD records where date of specimen collection was missing, optimal date of onset was used instead (further details on this variable below). Records with a specimen collection date on or after the date of death were considered post-mortem specimens and were excluded from these analyses. As the cohort consisted of all births from 1996 to 2012, this study includes notification and laboratory data for children up to 16 years of age.

### Definitions and linkage rules

Aboriginal children were identified using the ‘Get Our Story Right’ variable, which was provided through the WA Data Linkage System [12]. Geographical region was assigned using the residential postcode at the time of birth. The same child in PathWest and WANIDD datasets was identified using a study-specific personal identifier assigned through the WA Data Linkage System [3].

Date of specimen collection was used to identify WANIDD records with a corresponding PathWest record. Approximately 19.4% of all WANIDD records of any notifiable disease pertaining to the Triple I cohort had missing date of specimen collection. For these cases, optimal date of onset, which is a derived date variable calculated by WANIDD, was used instead. Optimal date of onset was calculated in hierarchical order based on date of onset, date of specimen collection, date of clinical notification and date of receipt of notification (personal communication, C Giele). If a date of onset was listed for a particular notification, this was used as the optimal date of onset. Date of receipt of notification was only used as the optimal date of onset if all other date variables were missing. Age of the child was calculated using the date of specimen collection and date of birth.

#### Influenza

Notification to WANIDD is mandatory for any laboratory-proven case of influenza infection detected in a respiratory specimen or serum by culture, polymerase chain reaction (PCR) and antigen testing or by serology [13]. Equivocal results were coded as not detected. Respiratory specimens were defined as nasal, sputum, throat, tracheal, lung or bronchial samples. Influenza became notifiable in 2001 [8]; hence cases were restricted to those detected between 2001 and 2012. Consistent with the WANIDD definition of duplicates, notifications or laboratory detections for the same child up to 8 weeks (56 days) from the date of initial specimen collection were considered duplicates unless a different subtype of influenza was detected. All duplicates were excluded from the following analyses.

#### Invasive pneumococcal disease (IPD)

An IPD case was defined as detection of *S. pneumoniae* by culture or PCR from a normally sterile site [13] including blood, cerebrospinal fluid or pleural fluid specimens. Detections from other sterile sites (e.g. joint fluid) were excluded for this study. Equivocal results were coded as not detected. As with influenza, IPD became notifiable in WA from 2001 onwards [8], hence, cases were restricted to those in 2001–2012. As per current rules applied to the WANIDD database, unless a different invasive serotype was identified, any subsequent records of IPD for the same person recorded on either WANIDD or PathWest were considered duplicates and excluded from these analyses.

#### Pertussis

Unlike influenza and IPD, notification is required for both laboratory-confirmed and probable cases of pertussis. Laboratory confirmation of pertussis was defined as the detection of *B. pertussis* by culture, PCR or serology [13]. PCR testing was in use throughout the study period and equivocal results were coded as not detected. Probable cases were defined as the presence of clinical (e.g. coughing illness for 2 or more weeks) together with epidemiological evidence of infection (e.g. epidemiological link to a laboratory-confirmed case) [13]. Clinicians are encouraged to submit a notification if they suspect an individual to have pertussis based on these criteria. WANIDD data for both laboratory-confirmed and probable cases were included in the following analyses. All PathWest cases that met the definition for laboratory confirmation of pertussis from 2000 onwards were included in the analyses. WANIDD or PathWest records for the same person up to a year (365 days) from the date of initial specimen collection were considered duplicates and excluded from the analyses.

### Statistical analyses

After extracting data from PathWest and WANIDD that met the criteria for notification, we compared the demographic factors of cases from the PathWest dataset to those from the WANIDD dataset. We then combined both datasets to calculate the total number of unique influenza, IPD and pertussis cases as well as the number of cases that were recorded in the WANIDD dataset only, the PathWest dataset only, or in both datasets. We then estimated and compared the incidence/notification rates (hereafter referred to as incidence rates) using person-time-at-risk as the denominator. Person-time was calculated using date of birth, death and the end of the study period. As the highest burden of respiratory infections are in children aged less than 5 years [14], only incidence rates for these children are presented by year of specimen collection using data from WANIDD alone and data from both datasets. Data cleaning and analyses were performed in IBM SPSS version 22 and 23. Exact 95% confidence intervals (CI) and incidence rate ratios (IRR) were calculated using EpiBasic [15].

## Results

Overall, between 2001 and 2012, there were 4885 influenza cases and 342 IPD cases recorded in PathWest from children in the Triple I cohort. WANIDD recorded 5159 influenza cases and 502 IPD cases over the same period. Between 2000 and 2012, there were a total of 2850 pertussis cases from children in the Triple I cohort recorded in PathWest while WANIDD recorded 4361 pertussis cases.

Demographics of children with influenza or IPD recorded in PathWest and WANIDD were similar (Table 1). Among children with pertussis, although the age distribution in both WANIDD and PathWest were similar, older children accounted for a larger proportion of cases reported in WANIDD compared to PathWest (Table 1). The majority of pertussis cases documented on WANIDD (*n* = 4247, 97.4%) were reported as laboratory-confirmed pertussis cases.

**Table 1:** Demographics of influenza, pertussis and IPD cases in WANIDD and PathWest datasets in 2000–2012

	Influenza (2001–2012)				IPD (2001–2012)				Pertussis (2000–2012)			
	PathWest (*N* = 4885)		WANIDD (*N* = 5159)		PathWest (*N* = 342)		WANIDD (*N* = 502)		PathWest (*N* = 2850)		WANIDD (*N* = 4361)	
	*n*	%	*n*	%	*n*	%	*n*	%	*n*	%	*n*	%
Male	2687	55.0	2849	55.2	185	54.1	285	56.7	1412	49.5	2154	49.4
Aboriginal	708	14.5	698	13.5	82	24.0	133	26.4	253	8.9	314	7.2
Age at specimen collection												
<6 months	328	6.7	309	6.0	31	9.1	36	7.2	391	13.7	434	10.0
6–12 months	433	8.9	416	8.1	48	14.0	67	13.3	129	4.5	166	3.8
12–23 months	649	13.3	629	12.2	94	27.5	145	28.8	216	7.6	301	6.9
2–4 years	1318	27.0	1363	26.4	112	32.7	158	31.4	537	18.8	834	19.1
5–9 years	1360	27.8	1513	29.3	43	12.6	74	14.7	901	31.6	1447	33.2
10–16 years	797	16.3	929	18.0	14	4.1	23	4.6	676	23.7	1179	27.0
Year of specimen collection												
2000^a^	N/A	-	N/A	-	N/A	-	N/A	-	16	0.6	19	0.4
2001	79	1.6	74	1.4	50	14.6	72	14.3	35	1.2	41	0.9
2002	328	6.7	300	5.8	43	12.6	66	13.1	57	2.0	67	1.5
2003	397	8.1	337	6.5	28	8.2	42	8.3	32	1.1	50	1.1
2004	82	1.7	51	1.0	37	10.8	47	9.3	261	9.2	361	8.3
2005	195	4.0	182	3.5	18	5.3	22	4.4	98	3.4	104	2.4
2006	100	2.0	72	1.4	8	2.3	22	4.4	39	1.4	37	0.8
2007	295	6.0	295	5.7	10	2.9	21	4.2	10	0.4	9	0.2
2008	286	5.9	299	5.8	18	5.3	26	5.2	69	2.4	89	2.0
2009	1008	20.6	1170	22.7	23	6.7	33	6.6	173	6.1	214	4.9
2010	382	7.8	406	7.9	34	9.9	49	9.7	321	11.3	452	10.4
2011	407	8.3	435	8.4	44	12.9	67	13.3	1042	36.6	1692	38.8
2012	1326	27.1	1538	29.8	29	8.5	36	7.2	697	24.5	1226	28.1
Region at birth												
Metropolitan	3697	75.7	3972	77.0	248	72.5	344	68.4	1894	66.5	3018	69.2
Rural	602	12.3	625	12.1	33	9.6	60	11.9	676	23.7	988	22.7
Remote	576	11.8	550	10.7	61	17.8	99	19.7	276	9.7	347	8.0

*IPD* invasive pneumococcal disease, *WANIDD* Western Australian Notifiable Infectious Diseases Database, *PathWest* PathWest Laboratory Medicine Western Australia Database. Aboriginal refers to Aboriginal and Torres Strait Islander people. Percentages may not equal 100 due to rounding and missing data

^a^Influenza and IPD data restricted to 2001–2012

**Fig. 1:**
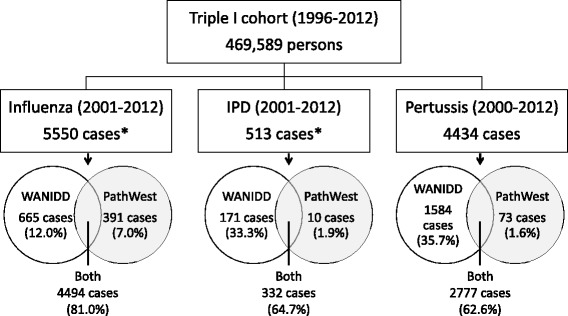
Number of influenza, IPD and pertussis cases that were found in each datasets. IPD = invasive pneumococcal disease; WANIDD = Western Australian Notifiable Infectious Diseases Database; PathWest = PathWest Laboratory Medicine Western Australia Database. *Influenza and IPD data restricted to 2001–2012

When WANIDD and PathWest data were combined, there were 5550 unique influenza cases, 513 IPD cases and 4434 pertussis cases from children in the Triple I cohort (Fig. 1). Using the WANIDD definition of duplicates, a total of 133 influenza cases, 17 IPD cases and 133 pertussis cases had duplicate records for the same episode of infection. Less than 2% of all pertussis (1.6%, 95% CI = 1.3–2.1%) and IPD cases (1.9%, 95% CI = 0.9–3.6%) were only recorded in the PathWest dataset (Fig. 1). In contrast, cases that were only recorded on PathWest accounted for 7.0% (95% CI = 6.4–7.8%) of all influenza cases (Fig. 1).

**Fig. 2:**
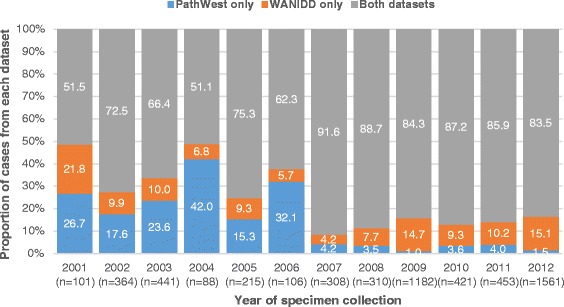
Proportion of influenza cases recorded in each dataset by year of specimen collection (2001–2012). WANIDD = Western Australian Notifiable Infectious Diseases Database; PathWest = PathWest Laboratory Medicine Western Australia Database. Percentages may not equal to 100 due to rounding

Notification of influenza cases to WANIDD improved over time with at least 95% of all cases being captured by WANIDD from 2007 onwards (Fig. 2). Similarly, the proportion of pertussis cases captured by WANIDD improved from 95.0% of all cases in 2001 to almost all cases (99.1%) in 2012 (Additional file 1: Figure S1). However, there was an increase in the proportion of pertussis cases captured by PathWest alone in 2006 and 2007, although the number of cases were small (<50 in each year). The WANIDD dataset captured nearly all IPD cases during the study period, with the exception of 2008, where 7.1% of cases were only recorded on PathWest. However, this represented only a small number of IPD cases (*n* < 5; Additional file 2: Figure S2).

**Fig. 3:**
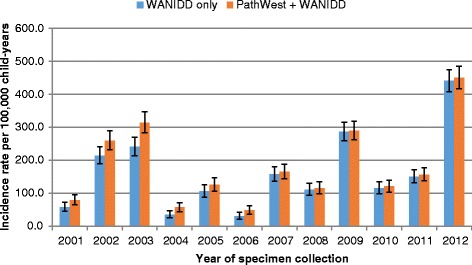
Influenza incidence rates in children aged less than 5 years by year of specimen collection. WANIDD = Western Australian Notifiable Infectious Diseases Database; PathWest = PathWest Laboratory Medicine Western Australia Database

**Fig. 4:**
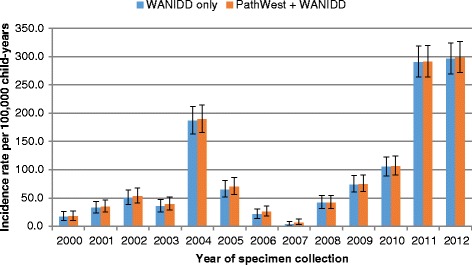
Pertussis incidence rates in children aged less than 5 years by year of specimen collection. WANIDD = Western Australian Notifiable Infectious Diseases Database; PathWest = PathWest Laboratory Medicine Western Australia Database

**Fig. 5:**
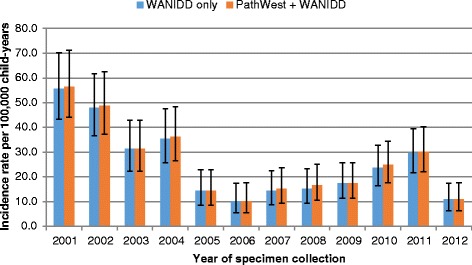
IPD incidence rates in children aged less than 5 years by year of specimen collection. WANIDD = Western Australian Notifiable Infectious Diseases Database; PathWest = PathWest Laboratory Medicine Western Australia Database

In children aged less than 5 years, the overall incidence rate of influenza was 168 per 100,000 child-years in 2001–2012 when using data from WANIDD alone. Using data from both datasets yielded a 10% increase in the overall incidence rate to 186 per 100,000 child-years (IRR = 1.1, 95% CI = 1.1–1.2), with the most marked difference in 2003 (Fig. 3). Overall incidence rates of pertussis was similar (IRR = 1.0, 95% CI = 1.0–1.1) when using data from both datasets (110 per 100,000 child-years) compared to data from WANIDD alone (107 per 100,000 child-years) with minimal difference over the study period (Fig. 4). Likewise, incidence rates of IPD were similar across all years when using data from either WANIDD only or both datasets (Fig. 5).

## Discussion

Respiratory infections are a major cause of hospitalisation in young children. Accurate, complete and reliable measures of disease burden, particularly with laboratory confirmation, are essential to guide the control of infectious diseases and assess the impact of prevention programs such as vaccination. Using a birth cohort and linked data, we described the discrepancies in the number of influenza, IPD and pertussis cases recorded in the WANIDD and PathWest datasets as well as the effect of these discrepancies on estimates of incidence rates. We anticipated that all PathWest cases would be captured in WANIDD but found additional cases of influenza, IPD and pertussis that were only documented in the PathWest dataset. While over 98% of IPD and pertussis cases were captured by WANIDD, it failed to capture 7% of all influenza cases. Incidence of influenza in children aged less than 5 years increased by 10% as a result of using data from both datasets compared to using only data from WANIDD.

Despite having an automated reporting system at PathWest to report notifiable pathogens to WANIDD, the WANIDD dataset failed to capture between 2 and 7% of notifiable disease cases. Our investigation uncovered two reasons why this occurred. Firstly, the automated notification system did fail on occasion, particularly in the early years. Secondly, influenza cases that were only detected by antigen detection were not electronically notified by the laboratory. Reports on the test results of these cases requested that the heath care provider notify the case, which did not reliably happen. This emphasises the importance of direct notification by laboratories in achieving high notification rates.

Discrepancies between the two datasets were greatest for influenza but this decreased over time. Both influenza and IPD became notifiable in 2001, with the laboratories developing automatic notification systems shortly thereafter. A decrease in automatic notification failures over time is consistent with development, implementation and audit of automated notifications. However, we observed an increase in the proportion of pertussis cases that were only recorded in the PathWest dataset in 2007. As new tests are introduced or refined over time, changes in the way these tests are coded could lead to errors in the automated reporting system. As PathWest Laboratories underwent significant changes to its database in 2006, both of these factors may have contributed to the sudden changes in notification patterns for influenza and pertussis in 2006–2007. In addition, a manufacturing error in the cut-off titres for serological testing for pertussis that was reported in 2006 [16] could have also contributed to the discrepancies between the two datasets during this period.

As notification data help to shape national responses to seasonal diseases like influenza [17] and guide immunisation policy [18], using notification data alone will likely underestimate the incidence of laboratory-confirmed infections as we have observed in this study. Similar issues with other surveillance models have been reported internationally [19]; we can only speculate if the issues presented here are applicable to other pathology providers and jurisdictions around Australia. We would welcome validation of these findings elsewhere using similar methodologies, although it may be more complicated if multiple pathology providers service substantial portions of the same locale. In the meantime, we would suggest using multiple data sources alongside statistical models or other methods, as appropriate, to generate future estimates of laboratory-confirmed infections wherever possible.

These results have been generated using linked data on the Triple I cohort. As such, it represents only a subset of the total population with a number of tests and notifications occurring in children outside the cohort. Furthermore, children exhibiting milder disease symptoms, particularly for influenza [20], may not be tested for the associated pathogen. It is estimated that PathWest contributes 64% of influenza, 44% of IPD and 34% of pertussis laboratory-confirmed notifications among WA residents (personal communication, C Giele). However, using record linkage provides a unique opportunity to externally validate notification data.

A further limitation of this study was missing data, particularly for key variables such as date of specimen collection. This may have flow on effects whereby relevant records were not extracted, which has occurred in previous studies [21], and associated PathWest records may not be identified. Due to the administrative nature of these datasets, investigators have little control over the quality and completeness of individual variables in each dataset. As the data are de-identified, we are unable to look up individual records to determine why a particular infection episode in one dataset was not found in the other. While this study is not an exhaustive investigation into the reasons for discrepancies between the two datasets, nevertheless, it provides a descriptive overview of areas for further investigation.

## Conclusions

To our knowledge, this is the first study that directly validates infectious disease notification data with routinely collected laboratory data for notifiable respiratory infections extracted through population-based record linkage. We identified discrepancies in the number of notifiable infectious diseases reported in WANIDD compared to PathWest, most notably for influenza. While we conducted investigations into the reasons for these discrepancies, the scope of investigations were limited by privacy restrictions with using linked data.

Periodic validation of passive surveillance systems like WANIDD, help identify reporting gaps and instil confidence in public health policy recommendations made from these data. A strength of this study is consultation with staff at PathWest and WANIDD to help verify our findings and ensure both laboratory and notification data were interpreted appropriately. Future studies describing the burden of laboratory-confirmed infections would benefit from the use of multiple data sources where feasible.

## Additional files

Additional file 1: Figure S1. Proportion of pertussis cases recorded in each dataset by year of specimen collection (2000–2012). WANIDD = Western Australian Notifiable Infectious Diseases Database; PathWest = PathWest Laboratory Medicine Western Australia Database. Percentages may not equal to 100 due to rounding. (DOCX 31 kb)

Additional file 2: Figure S2. Proportion of IPD cases recorded in each dataset by year of specimen collection (2001–2012). WANIDD = Western Australian Notifiable Infectious Diseases Database; PathWest = PathWest Laboratory Medicine Western Australia Database. Percentages may not equal to 100 due to rounding. (DOCX 30 kb)

## Abbreviations

CI: Confidence intervals
IPD: Invasive pneumococcal disease
IRR: Incidence rate ratio(s)
PathWest: PathWest Laboratory Database
PCR: Polymerase chain reaction
WA: Western Australia
WANIDD: Western Australian Notifiable Infectious Diseases Database
