# Supplementary material for: Using record linkage to validate notification and laboratory data for a more accurate assessment of notifiable infectious diseases
Source: BMC Med Inform Decis Mak. 2017 Jun 17;17:86. doi: 10.1186/s12911-017-0484-7 (PMC5473994; doi:10.1186/s12911-017-0484-7)
Supplement: Supplementary file 1 [file 12911_2017_484_MOESM1_ESM.docx]

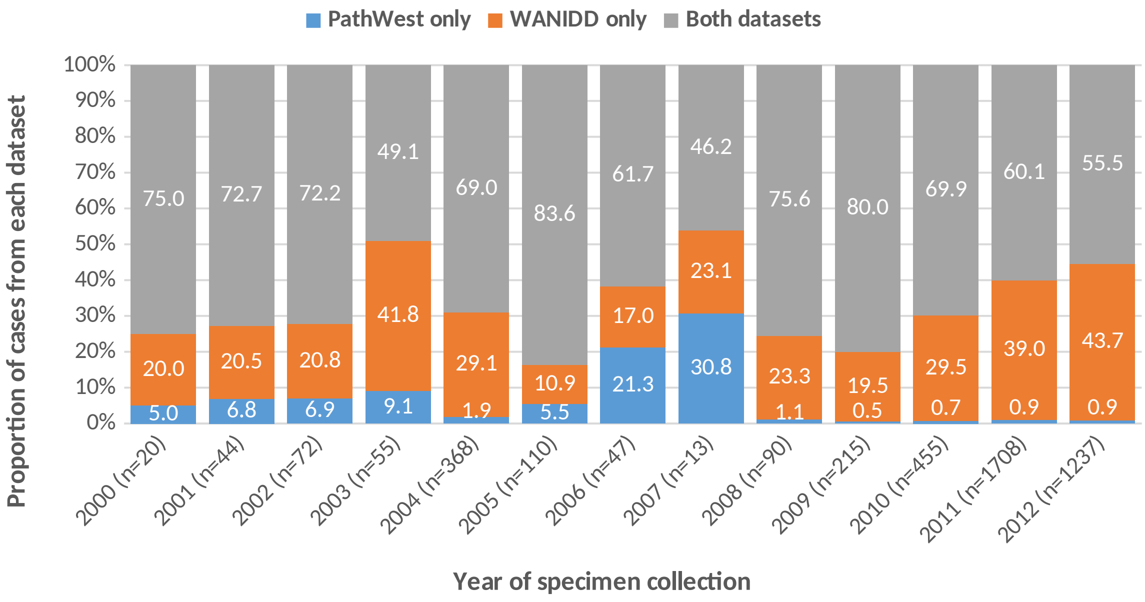
PathWest only
WANIDD only
Both datasets
100%
90%
80%
70%
60%
50%
40%
30%
20%
10%
0%
Proportion of cases from each dataset
75.0
72.7
72.2
49.1
69.0
83.6
61.7
46.2
75.6
80.0
69.9
60.1
55.5
20.0
20.5
20.8
41.8
29.1
10.9
17.0
23.1
23.3
19.5
29.5
39.0
43.7
5.0
6.8
6.9
9.1
1.9
5.5
21.3
30.8
1.1
0.5
0.7
0.9
0.9
2000 (n=20)
2001 (n=44)
2002 (n=72)
2003 (n=55)
2004 (n=368)
2005 (n=110)
2006 (n=47)
2007 (n=13)
2008 (n=90)
2009 (n=215)
2010 (n=455)
2011 (n=1708)
2012 (n=1237)
Year of specimen collection
