# Supplementary material for: Using record linkage to validate notification and laboratory data for a more accurate assessment of notifiable infectious diseases
Source: BMC Med Inform Decis Mak. 2017 Jun 17;17:86. doi: 10.1186/s12911-017-0484-7 (PMC5473994; doi:10.1186/s12911-017-0484-7)
Supplement: Supplementary file 2 [file 12911_2017_484_MOESM2_ESM.docx]

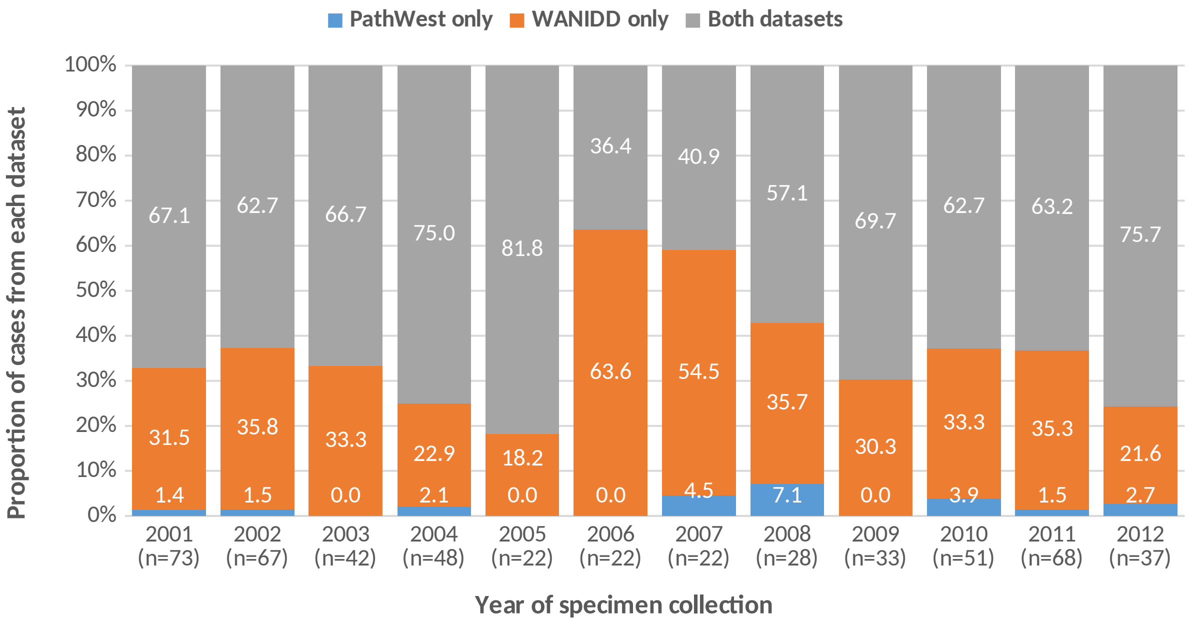
PathWest only
WANIDD only
Both datasets
Proportion of cases from each dataset
100%
90%
80%
70%
60%
50%
40%
30%
20%
10%
0%
67.1
62.7
66.7
75.0
81.8
36.4
40.9
57.1
69.7
62.7
63.2
75.7
31.5
35.8
33.3
22.9
18.2
63.6
54.5
35.7
30.3
33.3
35.3
21.6
1.4
1.5
0.0
2.1
0.0
0.0
4.5
7.1
0.0
3.9
1.5
2.7
2001 (n=73)
2002 (n=67)
2003 (n=42)
2004 (n=48)
2005 (n=22)
2006 (n=22)
2007 (n=22)
2008 (n=28)
2009 (n=33)
2010 (n=51)
2011 (n=68)
2012 (n=37)
Year of specimen collection
